# Effect of spray-dried porcine plasma and plasma hydrolysate on the health and performance of mycotoxin-challenged piglets at weaning

**DOI:** 10.1007/s11250-026-04923-z

**Published:** 2026-02-26

**Authors:** Lucieli Kamila Muller, Diovani Paiano, Rafael Domingos Augusto Rofino, Sofia Contini, Tatiane L. Esposito, Maria Eduarda de Costa, Gilnei Bruno da Silva, Daiane Manica, Margarete Dulce Bagatini, Eduardo M. da Gloria, Aleksandro S. da Silva

**Affiliations:** 1 Programa de Pós-graduação em Bioquímica e Biologia Molecular, Universidade do Estado de Santa Catarina (UDESC), Lages, Brasil; 2 Departamento de Zootecnia, UDESC, Chapecó, Brasil; 3 Programa de Pós-graduação em Zootecnia, UDESC, Chapecó, Brasil; 4 Centro de Ciências da Saúde, Universidade Federal Fronteira Sul, Chapecó, Brasil; 5 Department of Biological Science, Luiz de Queiroz” College of Agriculture, University of São Paulo, Piracicaba, Brasil; 6 Postgraduate Program in Biochemistry, Federal University of Santa Catarina, Florianópolis, Brazil

**Keywords:** Mycotoxin, Pig farming, Spray-dried plasma, Hydrolyzed plasma

## Abstract

This study aimed to evaluate the effects of Spray-dried porcine plasma (SDPP) and hydrolysate plasma (HP) in the diets of post-weaning piglets challenged with mycotoxins (aflatoxin and fumonisin). The experiment lasted a total of 35 days, divided into three stages: pre-nursery I (d1-7), pre-nursery II (d8-14) and initial (d15-35). Seventy-two entire male piglets (26 days old) distributed in six treatments were tested, consisting of diets: T1 control (without SDPP, HP, and mycotoxins); T2 - with combined mycotoxins (300 ppb aflatoxin + 8.0 ppm fumonisin); T3 - with HP (pre-1–6%; pre-2–4%); T4 - with HP (pre-1–6%; pre-2–4%) + mycotoxins (300ppb aflatoxins + 8ppm fumonisin); T5 - with SDPP (pre-1–6%; pre-2–4%); T6 - with SDPP (pre-1–6%; pre-2–4%) + mycotoxins (300ppb aflatoxins + 8ppm fumonisin). Mycotoxins had a negative effect on performance, reducing body weight, and average daily gain (ADG) at days 15 to 35, in addition to increasing the levels of reactive oxygen species (ROS), lipid peroxidation (TBARS), myeloperoxidase (MPO) and protein thiols (PSH) (*p* < 0.05). While SDPP increased body weight, ADG and daily feed intake (DFI), in addition to presenting an antioxidant effect (*p* < 0.05). Intake of HP increased DFI (d1 to 7; and d8 to 14), but induced an increase in ROS (d35) and TBARS at day 14 (*p* < 0.05). The addition of SDPP and HP to the mycotoxin-contaminated diet was unable to minimize or prevent the disorders caused by aflatoxin and fumonisin. The mycotoxins caused subclinical intoxication with alterations in sensitive biomarkers of cellular damage, and oxidative stress, in addition to compromising animal performance. SDPP group provided greater performance than HP group. While HP, despite increasing feed intake, appears to stimulate pro-oxidant variables. According to our results, appear that SDPP and HP have different properties, which resulted in different results for various performance and health biomarkers in piglets under mycotoxin contamination.

**Supplementary information:**

The online version contains supplementary material available at https://doi.org/10.1007/s11250-026-04923-z.

## Introduction

Pig production involves several challenges, some of which are more pronounced at certain stages, such as weaning (Maciag et al. 2022). Weaning involves switching from a liquid to a solid diet, environmental changes, and the need for resocialization, which infers changes in the morphology and physiology of the gastrointestinal tract, alterations in the intestinal microbiota, and reduced immunity (Tang et al. 2022). The challenge resulting from the combination of these stressors can be further exacerbated by the presence of mycotoxins (Kipper et al. 2020). These mycotoxins are produced by filamentous fungi and can be present in feed, especially those from plant-based products (Kolawole et al. 2024). Mycotoxins suppress the immune system, opening the door to opportunistic pathogens, and contributing to gastrointestinal problems and nutrient malabsorption, resulting in losses in performance and productivity (Liew et al. 2018).

Nutritional strategies are used to mitigate the harmful effects of weaning (Huting et al. 2021) and mycotoxins (Müller et al. 2018a). Spray-dried porcine plasma (SDPP) is considered a functional food, used occasionally during piglet weaning. It has demonstrated interesting beneficial effects, such as increased feed intake and weight gain (Balan et al. 2021), reduced diarrhea incidence (Müller et al. 2018a), and antioxidant and anti-inflammatory effects (Müller et al. 2018a, 2019). According to Müller et al. (2018b), when SDPP is added to a diet contaminated with mycotoxins, although SDPP does not interact with mycotoxins and both have isolated effects, SDPP contributes to the health status and improved performance of piglets. However, the mechanism of action of SDPP, through which these beneficial effects are provided, is still unclear. One of the arguments being used is the narrative surrounding the presence of immunoglobulins in SDPP, which represent around 20% of the protein fraction (Balan et al. 2021). Although are not absorbed intact after 48 hours of life (Heim et al. 2015), they could have some local action in the intestine, resulting in greater intestinal health, better mucosal integrity with reduced permeability to pathogens, and reduced incidence of diarrhea (Zhang et al. 2015). It is known that in pigs, the first phase of enzymatic chemical digestion occurs in an acidic environment in the stomach, where proteins are denatured and unfolded, subsequently broken down by enzymatic action, resulting in free amino acids and small peptides capable of being absorbed (Sakomura 2014). Once denatured, the protein loses its activity, which in the case of immunoglobulins would be its defense against pathogens. Therefore, the protective effect attributed to SDPP from immunoglobulins becomes an unfounded argument when considering the digestive physiology of animals. In order to understand the mechanism, the idea of hydrolyzing plasma arises, a process that would maintain only the nutritional effect, without the presence of functional globulins.

To determine whether SDPP effect and its functions as a functional feed, or whether the benefits are solely nutritional, stemming from high biological value protein, with a high proportion of essential amino acids and an absence of antinutritional factors, are needed. Thus, the objective of the present study was to evaluate the effects of both SDPP and hydrolysate plasma (HP) in the diet of post-weaning piglets challenged with mycotoxins on performance, immune response, antioxidant activity and health status.

## Material and methods

### Spray-dried porcine plasma (SDPP) and hydrolyzed plasma (HP)

The SDPP (AP 920®, APC) used contained at least 78% crude protein, a maximum of 10% mineral matter, a minimum of 0.5% ether extract, and 92% dry matter. The HP (Pepteiva®, APC) contained a minimum of 70% crude protein, a maximum of 20% mineral matter, a minimum of 2.6% ether extract, and 91% dry matter.

### Mycotoxin: production and quantification methodology

The mycotoxin production used in this study was previously detailed by Muller et al. (2017). Aflatoxins were obtained by rice fermentation, using a temperature-controlled method with constant agitation, and the *Aspergillus nomius* strain was used for mycotoxin production. Fumonisins were obtained from corn grain fermentation, and the *Fusarium verticillioides* strain was used for mycotoxin production. The contamination levels proposed in our study were achieved in the diets by adding these concentrates with mycotoxins.

For mycotoxins analyses samples of feed were ground to < 0.85 mm and one gram of the ground material was transferred to 50 mL test tube. It was added 10 mL of ultrapure water and 10 mL of acetonitrile/acetic acid (CH3CN:CH3COOH) [99.5:0.5, v/v] and the test tube was placed in a mechanical shaker for 10 min. A mixture of 4 g of MgSO4 and 1 g of NaCl was added and the tube was vigorously hand-shaken for 10 s. The solution was them centrifuged for 15 min at 5.000 ×g, at 25 ◦C and 2.5 mL of supernatant was transferred to capped glass test tube where 2.5 mL of hexane was added. The solution was shaken for 2 h and then centrifuged at 1.000 ×g, at 20 ◦C for 1 min. From lower phase (acetonitrile) 1 mL was withdraw and dried with Nitrogen (N2) stream at 40 ◦C. The reconstitution was performed with 75 µL of methanol in ultrasonic bath for 10s and 10s in test tube mixer after adding 75 µL of ultrapure water. After centrifugation for 10 min at 14.000 ×g 60 µL was withdraw and transferred to vial where 140 µL of ultrapure water was added. Ten microlitres were injected in chromatographic system.

Detection and quantification of mycotoxins were performed with high-performance liquid chromatography coupled with tandem mass-spectrometry (LC/MS/MS). Chromatographic separation was carried out using Acqulty UPLC System (Waters, Milford, Massachusetts, EUA) equipped with 100 × 2.1 mm, 1.7 µm Acquity UPLC BEH C18 column, (Waters, Milford, Massachusetts, EUA). The column was maintained at 40 ◦C and the injection volume was 10 µL. The mobile phase consisted of 0.1% formic acid in water(A), and 0.1% formic acid in acetonitrile (B). The acetonitrile (B) concentration was raised gradually from 10% to 90% within 12 min, brought back to the initial conditions at 0,1 min, and allowed to stabilize for 3 min. The mobile phase was delivered at a flow rate of 0.4 mL/min. The LC system was coupled with Xevo TQS tandem mass spectrometer (Waters, Milford, Massachusetts, EUA), equipped with a turbo-ion electrospray (ESI) ion source. The mass-spectrometer was operated in scheduled multiple reaction monitoring (MRM) in positive mode. The data acquisition of mass spectrometer are showed in Table S1.

The contamination level of the fumonisin-contaminated material was 1626.70 ppm of fumonisin B1, equivalent to 6.0 grams; and 289.40 ppm of aflatoxin B1, equivalent to 0.222 grams. To achieve a final dietary contamination level of approximately 8.0 ppm of fumonisin B1, 491.80 g/100 kg of feed was added. The contamination level of the aflatoxin-contaminated material was 289.40 ppm of Aflatoxin B1, equivalent to 0.222 grams. To achieve a final dietary contamination level of approximately 300 ppb of Aflatoxin B1, 103.7 g/100 kg of feed was added. These contamination levels were used to replicate similar conditions of a previous experiment conducted by our study group (Müller et al. 2017), in which these levels were defined, as they can cause harmful effects, but may be encountered in natural contamination (Weaver et al. 2014).

### Animals and facilities

The experiment was conducted at Experimental Farm of Universidade do Estado de Santa Catarina, located in Guatambu, Santa Catarina State, Brazil. Seventy-two commercially weaned male piglets were (26±1 days old) distributed in a completely randomized design (CRD) in a 2 × 3 factorial arrangement, totaling six treatments, with six replicates each and two animals per experimental unit. The experiment lasted 35 days.

The animals were housed in pens with perforated PVC floors, equipped with automatic drinkers and manually replenished trough feeders, each spaced 15 cm apart. The temperature was controlled by a thermostat, which activated the heaters whenever it reached the programmed minimum temperature of 28 °C and deactivated it at 29 °C, reducing the temperature by 1 °C per week. The animals were weighed at the beginning, middle, and end of the experiment. Daily feed intake was measured, and daily weight gain and feed conversion were calculated at the end of the experimental phase. The pen with 2 animals each was considered an experimental unit.

### Experimental design and diets

Six treatments were tested, consisting of diets: T1 - control without SDPP, HP, and mycotoxins; T2 - with combined mycotoxins (300 ppb aflatoxin + 8.0 ppm fumonisin); T3 - with HP (pre-1–6%; pre-2–4%); T4 - with HP (pre-1–6%; pre-2–4%) and with mycotoxins (300 ppb aflatoxin + 8 ppm fumonisin); T5 - with SDPP (pre-1–6%; pre-2–4%); T6 - with SDPP (pre-1–6%; pre-2–4%) and with mycotoxins (300 ppb aflatoxin + 8 ppm fumonisin). It is important to emphasize that the pen (with 2 piglets) was defined as the experimental unit; both in the performance analyses and in the blood analysis.

The experimental diets (Table 1) were formulated according to the nutritional requirements available in the Brazilian Swine and Poultry Tables, indicated by Rostagno et al. (2017). They were provided ad libitum, with three different diets (pre-diet 1 - day 1 to 7; pre-diet 2 - day 8 to 14; starter diet - day 15 to 35), with a total of 35 days of experimentation. The starter diet did not contain SDPP or HP, but maintained the mycotoxin levels (300 ppb aflatoxin + 8 ppm fumonisin) in the treatments that received mycotoxins in the previous phases. The mycotoxin doses were determined aiming to provide subclinical toxicity, as already seen in previous works by the study group (Müller et al. 2017), but at the same time to be doses that could be found in field situations, natural contamination, as described by Weaver et al. (2014). Mycotoxin levels (aflatoxin and fumonisin) in the feed were measured using chromatography analysis as described previously in inoculum.

**Table 1:** Diet ingredients and calculated centesimal composition of the diets. Cont: control diet; Myco: control diet with mycotoxins; HP: diet with plasma hydrolysate; HP+Myco: diet with plasma hydrolysate and mycotoxins; SDPP: diet with SDPP; SDPP+Myco: diet with SDPP plus mycotoxins and starter diet

	Pre-Starter I (1-7d)						Pre-Starter II (8-14d)						Initial (15-35d)	
Rations	Controls (C)		Hydrolyzed Plasma (HP)		Spray dried plasma (SDPP)		Controls (C)		Hydrolyzed Plasma (HP)		Spray dried plasma (SDPP)			
Mycotoxins	Neg (-)	Pos (+)	Neg (-)	Pos (+)	Neg (-)	Pos (+)	Neg (-)	Pos (+)	Neg (-)	Pos (+)	Neg (-)	Pos (+)	Neg (-)	Pos (+)
Corn, %	37.2	36.9	40.9	40.9	42.1	42.1	53.6	53.2	55.6	55.6	56.8	56.8	63.0	62.5
Soybean meal, %	33.8	33.8	24.5	24.5	24.0	24.0	29.8	29.8	24.0	24.0	23.4	23.4	29.0	29.0
Whey Powder, %	15.0	15.0	15.0	15.0	15.0	15.0	7.50	7.50	7.50	7.50	7.50	7.50	-	-
Soybean Oil, %	0.30	0.30	0.30	0.30	0.30	0.30	0.30	0.30	0.30	0.30	0.30	0.30	-	-
Sugar, %	5.00	5.00	5.00	5.00	5.00	5.00	1.00	1.00	1.00	1.00	1.00	1.00	-	-
Bewi-Spray 99, %	3.84	3.84	3.34	3.34	3.05	3.05	3.21	3.21	2.98	2.98	2.65	2.65	3.18	3.180
Limestone, %	0.695	0.695	1.094	1.094	0.970	0.970	0.672	0.672	0.938	0.938	0.859	0.859	0.835	0.835
Salt, %	0.189	0.189	-	-	0.080	0.080	0.125	0.125	-	-	0.051	0.051	0.255	0.255
Dicalcium Phosp., %	1.035	1.035	0.505	0.505	0.700	0.700	1.134	1.134	0.780	0.780	0.910	0.910	1.120	1.120
Bicarbonate, %	0.355	0.355	-	-	0.030	0.030	0.370	0.370	0.113	0.113	0.155	0.155	0.342	0.342
Premix^a^, %	0.850	0.850	0.850	0.850	0.850	0.850	0.600	0.600	0.600	0.600	0.600	0.600	0.600	0.600
Lysine - HCl, %	0.538	0.538	0.314	0.314	0.320	0.320	0.575	0.575	0.422	0.422	0.434	0.434	0.580	0.580
DL-Methionine, %	0.262	0.262	0.216	0.216	0.180	0.180	0.233	0.233	0.200	0.200	0.179	0.179	0.220	0.220
L-Threonine, %	0.280	0.280	0.117	0.117	0.170	0.170	0.287	0.287	0.174	0.174	0.212	0.212	0.420	0.420
L-Tryptophan, %	0.047	0.047	0.017	0.017	0.010	0.010	0.050	0.050	0.030	0.030	0.030	0.030	0.050	0.050
L-Isoleucine, %	-	-	-	-	-	-	0.019	0.019	-	-	0.025	0.025	0.020	0.020
L-Valine, %	0.166	0.166	-	-	0.030	0.030	0.148	0.148	0.400	0.400	0.053	0.053	0.167	0.167
Contaminant AF^c^, %	-	0.100	-	0.100	-	0.100	-	0.100	-	0.100	-	0.100	-	0.100
Contaminant FM^b^, %	-	0.490	-	0.490	-	0.490	-	0.490	-	0.490	-	0.490	-	0.490
Phytase^d^, %	0.500	0.500	0.500	0.500	0.500	0.500	0.500	0.500	0.500	0.500	0.500	0.500	0.500	0.500
Banox^f^, %	0.100	0.100	0.100	0.100	0.100	0.100	0.100	0.100	0.100	0.100	0.100	0.100	0.100	0.100
Flavor Sucram, %	0.020	0.020	0.020	0.020	0.020	0.020	0.020	0.020	0.020	0.020	0.020	0.020	0.020	0.020
Spray dried plasma, %	-	-	-	-	6.00	6.00	-	-	-	-	4.00	4.00	-	-
Hydrolyzed Plasma, %	-	-	6.680	6.680	-	-	-	-	4.460	4.460	-	-	-	-
Inert, %	0.270	-	1.2	0.412	1.035	0.445	0.163	-	0.774	0.184	0.587	-	0.086	-
	Calculated composition^e^													
Calcium, %	0.75	0.75	0.75	0.75	0.75	0.70	0.70	0.70	0.70	0.70	0.70	0.70	0.70	0.70
Available P, %	0.38	0.38	0.38	0.38	0.38	0.30	0.35	0.35	0.35	0.35	0.35	0.35	0.30	0.30
Sodium, %	0.30	0.30	0.30	0.30	0.30	0.20	0.22	0.22	0.22	0.22	0.22	0.22	0.20	0.20
Chlorine, %	0.36	0.36	0.36	0.36	0.36	0.36	0.23	0.23	0.23	0.23	0.23	0.23	0.22	0.22
Met. Energy, %	3389	3389	3390	3390	3391	3391	3363	3363	3363	3363	3364	3364	3350	3350
Crude Protein, %	20.97	20.97	21.26	21.26	21.19	21.19	19.56	19.56	19.85	19.85	19.79	19.79	19.12	19.12
Digestible lysine, %	1.455	1.455	1.445	1.445	1.445	1.445	1.347	1.347	1.347	1.347	1.347	1.347	1.282	1.282
Digestible Met + Cis, %	0.810	0.810	0.810	0.810	0.810	0.810	0.754	0.754	0.754	0.754	0.754	0.754	0.730	0.730
Digestible Treonine, %	0.963	0.963	0.963	0.963	0.963	0.963	0.902	0.902	0.902	0.902	0.902	0.902	0.992	0.992
Digestible Tryptophan, %	0.280	0.280	0.280	0.280	0.280	0.280	0.256	0.256	0.256	0.256	0.256	0.256	0.243	0.243
Digestible Valine, %	1.017	1.017	1.053	1.053	1.019	1.019	0.930	0.930	0.930	0.930	0.930	0.930	0.922	0.922
Digestible Isoleucine, %	0.812	0.812	0.825	0.825	0.800	0.800	0.743	0.743	0.746	0.746	0.743	0.743	0.705	0.705
Aflatoxin, ppb		300		300		300		300		300		300		300
Fumonisin, ppm		8.00		8.00		8.00		8.00		8.00		8.00		8.00

Minimum guaranteed levels per kg of product: Cu 120 g, Iron 140 g, Iodine 1.20 g, Mn 50 g, Se 0.40 g, Zn 1 g, Folic Acid20 mg, Pantothenic Acid 25.0 mg, Biotin0.30 mg, Choline1,460 g, Niacin 370 mg, Vitamin A15,0 IU, Vitamin B126 mg/kg, Vitamin B12 (Min) 40. mg/kg, Vitamin B2 (Min) 5.20 mg/kg, Vitamin B6 (Min) 3.90 mg/kg, Vitamin D33,0 IU, Vitamin E160 IU, Vitamin K352 mg, Lysine 12 g, L – Tryptophan2 g, L – Valine 8 g, DL-Methionine3 g, Threonine 8 g, Phytase 5 FTU. ^b^ Contaminant Rice with 130 mg de aflatoxin/kg (AF). ^c^ Contaminant Corn with 110 mg de fumonisin/kg (FM). ^d^ Comercial phytase with 20K FTU/kg ^e^ Values calculated based on the nutritional composition proposed by Rostagno et al. (2024). ^f^ Bewi®—palm fat and lecithin

### Data and sample collection

The experiment lasted 35 days. The animals were weighed at the beginning of the experiment (day 1) and on days 7, 14, and 35 to calculate weight gain and average daily weight gain. Feed intake was measured by period, that is, between days 1–7, 8–14, and 15–35, when diet changes were made. Based on feed intake (FI) and weight gain, feed conversion (FCR) was calculated: FI/ADG.

On days 7, 14, and 35, the animals were fasted for six hours. Blood was then collected via vena cava puncture. Anticoagulant tubes were used for hematological analyses (complete blood count) and clot-retractor tubes were used to separate the serum used in biochemical/metabolic analyses. The restraint method followed that recommended by Moreno et al. (1997), with collection performed by a duly trained person with prior authorization from the ethics committee for experimentation (under protocol No. 2,431,250,923). The animal was restrained for a maximum of 30 seconds, and the sample was collected from the cranial vena cava using needles (25x70 mm) and vacuum tubes. Approximately 2.5 mL were collected in EDTA tubes for blood count analysis and approximately 2.5 mL in activator tubes for subsequent serum extraction for analysis of biochemical indicators and oxidative stress.

### Serum biochemistry

Serum levels of biochemical variables such as protein, albumin, cholesterol, glucose,, bilirubin, C-reactive protein were measured, as well as activities of amylase, alanine aminotransferase (ALT), alanine aspartate transferase (AST), alkaline phosphatase, creatine kinase, and cholinesterase were using commercial kits from Analisa®. Transferrin, IgA, and IgG were measured using commercial kits from Labtest® and automated equipment (Zybio EXC 200®). The analyses were performed in duplicate. The linearity, and methods of the commercial kits used here are presented in Table S2, as well as reference range for piglets.

### Complete blood count

Immediately upon arrival at the laboratory, hemoglobin, erythrocyte, total leukocyte, and hematocrit counts were performed using an electronic Equipvet device (model 3000®). The WBC margin was counted according to the procedure described. For the leukocyte differential, a blood smear was performed according to the technique of Feldman et al. (2000), stained with a Pantico rapid kit for counting under an optical microscope at 1000x magnification.

### Oxidative stress

The oxidative status variables evaluated in the blood serum were reactive oxygen spe-cies (ROS), thiobarbituric acid-reactive substances (TBARS), and myeloperoxidase ac-tivity (MPO). The analyses were performed in triplicate using specific biochemical methodologies. Color and fluorometric readers were prepared using Varioskan™ LUX (Thermo Scientific™). ROS formation was estimated using a fluorometric protocol established by Ali et al. (1992). Ten microliters of serum were soaked in 10 µL of 2‘,7’-dichlorofluorescein diace-tate (DCFH-DA, 7 μM) and 240 µL of PBS. After 30 min of incubation at 37 °C, the final product of DCFH-DA oxidation, the dichlorofluorescein (DCF), was measured. The fluorescence emission intensity was read at an emission wavelength of 525 nm and an excitation wavelength of 488 nm. The results were expressed as a percentage (%) of the fluorescence intensity relative to the control. Lipoperoxidation is a highly rapid reaction formed by the breakdown of polyunsaturated-rated fatty acids, which are usually measured by their products, mainly thiobarbituric acid-reactive substances (TBARS), among which malondialdehyde (MDA) is the primary product (Jentzsch et al. 1996). To evaluate this product, the reaction of thiobarbituric acid (TBA) with serum samples was used, which, in the presence of MDA, resulted in a pink product that could be read at 532 nm. Briefly, 20 µL of each sample was mixed with 55 µL distilled water, 100 µL orthophosphoric acid (0.2 M), and 25 µL TBA (0.1 M). Spectrophotometric readings were taken after 45 min of incubation at 37 °C. The results are expressed in nanomolar MDA/mL. Thiobarbituric acid-reactive substances (TBARS) were measured as described by Jentzsch et al. (1996). Protein thiols (PSH) were determined as described by Sedlak and Lindsay (1968), based on the reaction of sulfhydryl groups (−SH) with 5,5’-dithiobis-(2-nitrobenzoic) acid (DTNB). Myeloperoxidase (MPO) was measured by catalyzes the oxidative coupling of phenol and 4-aminoantipyrine (AAP), yielding a colored product, quinoneimine, with a maximum absorbance of 492 nm (Suzuki et al. 1983).

### Statistical analysis

With the results in hand, a descriptive analysis of the data was performed. The data were tested for normality and homogeneity of variance using the Shapiro-Wilk and Levene tests, respectively. Platelet counts and amylase and creatine kinase enzyme activity were not normalized; these data were transformed logarithmically to normalize the data. The other variables showed normal data. Therefore, the data meet all the requirements for parametric testing, as described below.

All data were analyzed using the SAS ‘MIXED’ procedure (SAS Inst. Inc., Cary, NC, USA; version 9.4), with the Satterthwaite approximation to determine the denominator degrees of freedom for the fixed effects tested. In the experimental model, treatment, sampling day, and the interaction between treatment and sampling day were used as fixed effects. The random effect was pen by treatment for all variables. The first-order autoregressive covariance structure was selected according to the lowest Akaike information criterion; where the only covariate used in this study was the average body weight of the pen at the beginning of the experiment (day 1); which reflected a degree of freedom of 5.

Means were compared using orthogonal contrasts at 5% significance. The first contrast (C1) represented the effect of the mycotoxin [Cont versus Myco]; the second contrast (C2): [HP versus HP+Myco]; the third contrast (C3): [SDPP versus SDPP+Myco]; the fourth contrast (C4): [Cont versus HP]; the fifth contrast (C5): [Cont versus SDPP]; the sixth contrast (C6): [Myco versus HP+Myco]; the seventh contrast (C7): [Myco versus SDPP+Myco); and the eighth contrast (C8): [HP versus SDPP]. The orthogonal contrast analysis allowed us to more clearly show the effects of mycotoxins and feeds (HP and SDPP), and also to verify whether the use of these foods could prevent or minimize the negative effects of the toxins, as already reported by Müller et al. (2018a).

The results were presented as mean and standard error (SEM) in tables and figures. Significance was defined as *p* ≤ 0.05.

## Results

In feed contaminated with mycotoxins, levels of aflatoxin (MYCO: 306 ppb; HP+MYCO: 302 ppb; SDPP+MYCO: 300 ppb) and fumonisin (MYCO: 7.87 ppm; HP+MYCO: 7.91 ppm; SDPP+MYCO: 7.95 ppm) were found. Despite the piglets having consumed these quantities of mycotoxins, no apparent clinical signs were observed; likewise, there was no piglet mortality in the experiment.

Body weight results are presented in Table 2. The effect of treatment, and treatment versus day interaction were observed. The treatment × day interaction occurred on day 35, highlighting the higher body weight of piglets in the SDPP group. Comparing the groups, we found that animals fed SDPP had higher body weight compared to CONT on days 14 and 35. Body weight was higher in the SDPP treatment on day 14, when compared to the control (*p* < 0.05). In contrast analysis, higher body weights were observed in piglets in the CONT, HP, and SDPP groups compared to those with mycotoxins on day 35. On the same date, higher body weights were observed in piglets in the SDPP group compared to CONT, SDPP+MYCO compared to MYCO, and SDPP compared to HP. On day 35, treatments containing mycotoxins showed lower body weight, demonstrating the cumulative detrimental effect of mycotoxins on performance (*p* < 0.05). Animals receiving SDPP in the pre-starter phases had higher body weight compared to the control at 35 days (*p* < 0.05), but HP group had no effect (*p* > 0.05). When comparing the MYCO and SDPP+MYCO treatments, animals that consumed SDPP beforehand had higher body weight (*p* < 0.05), which was not the case with the HP treatment. When comparing animals that consumed SDPP versus those that consumed HP in the first two weeks, the SDPP treatment showed higher body weight (*p* < 0.05).

**Table 2:** Effect of treatment on zootechnical variables of piglets in the nursery phase receiving diets contaminated by mycotoxins and dehydrated porcine plasma (SDPP) and hydrolyzed porcine plasma (HP)

Variable	CONT	MYCO	HP	HP+MYCO	SDPP	SDPP+MYCO	SEM	C1	C2	C3	C4	C5	C6	C7	C8
Body Weight, (kg)															
d1	7.37	7.37	7.38	7.37	7.42	7.37	0.10	0.97	0.98	0.94	0.97	0.95	0.98	0.95	0.95
d7	8.82	9.29	9.31	8.91	9.36	9.73	0.16	0.45	0.51	0.35	0.34	0.32	0.64	0.56	0.79
d14	11.6	12.3	12.8	11.8	13.4	12.7	0.26	0.25	0.22	0.38	0.21	0.03	0.67	0.85	0.57
d35	24.8	23.1	25.7	22.8	27.5	24.7	0.37	0.05	0.02	0.01	0.26	0.01	0.82	0.05	0.04
Weight Gain (kg/piglet/period)															
d1-7	0.207	0.274	0.276	0.22	0.278	0.337	0.02	0.19	0.22	0.08	0.21	0.2	0.23	0.07	0.98
d1-14	0.305	0.355	0.391	0.318	0.426	0.379	0.02	0.28	0.12	0.02	0.15	0.01	0.56	0.71	0.16
d1-35	0.498	0.449	0.524	0.441	0.575	0.495	0.01	0.31	0.01	0.01	0.27	0.05	0.92	0.35	0.05
d8-14	0.402	0.436	0.506	0.416	0.575	0.422	0.02	0.28	0.09	0.01	0.09	0.01	0.83	0.93	0.04
d14-35	0.627	0.513	0.613	0.524	0.674	0.573	0.02	0.01	0.05	0.12	0.56	0.31	0.91	0.43	0.05
Feed Intake (kg/piglet/period)															
d1-7	0.245	0.294	0.327	0.293	0.29	0.357	0.02	0.51	0.46	0.35	0.03	0.34	0.92	0.15	0.58
d1-14	0.368	0.41	0.462	0.409	0.478	0.458	0.02	0.47	0.41	0.68	0.04	0.01	0.95	0.93	0.85
d1-35	0.66	0.612	0.709	0.613	0.772	0.67	0.02	0.89	0.27	0.19	0.75	0.06	0.97	0.81	0.35
d8-14	0.491	0.525	0.596	0.525	0.665	0.558	0.02	0.55	0.63	0.59	0.17	0.01	0.96	0.26	0.11
d14-35	0.854	0.747	0.874	0.75	0.969	0.811	0.03	0.19	0.37	0.08	0.92	0.31	0.98	0.33	0.22
Feed Conversion (kg/kg)															
d1-7	1.183	1.072	1.184	1.331	1.043	1.059	0.06	0.58	0.34	0.95	0.97	0.49	0.12	0.91	0.55
d1-14	1.206	1.154	1.181	1.286	1.122	1.208	0.03	0.45	0.42	0.63	0.55	0.66	0.26	0.48	0.87
d1-35	1.325	1.363	1.353	1.39	1.342	1.353	0.01	0.87	0.85	0.89	0.91	0.92	0.9	0.94	0.94
d8-14	1.221	1.204	1.177	1.262	1.156	1.322	0.04	0.92	0.36	0.05	0.52	0.17	0.88	0.52	0.76
d14-35	1.362	1.456	1.425	1.431	1.437	1.415	0.03	0.44	0.95	0.92	0.78	0.73	0.96	0.93	0.96

Data were analyzed by orthogonal contrasts, with the probability (*p*-value) described within the table: C1 (Cont ×Myco), C2 (HP x HP+Myco), C3 (SDPP x SDPP+Myco), C4 (Cont ×HP), C5 (Cont ×SDPP), C6 (Myco ×HP+Myco), C7 (Myco ×SDPP+Myco), C8 (HP x SDPP). Note: Each group had 6 repetitions (*n* = 6), where the bay average was used. SEM = standard error

In the periods d1-14, d1-35, and d8-14, mycotoxins had an effect on reducing ADG in the SDPP+MYCO treatments compared to SDPP (Table 2). The same effect was observed from period 1–35 in the HP+MYCO treatment compared to HP. On these same days, a higher ADG was observed for SDPP compared to the control. The effect of mycotoxins on ADG reduction continued on days 8–14 in the MYCO and HP+MYCO treatments. SDPP group showed higher ADG than HP on days 1–35, 8–14, and 14–35 (*p* < 0.05). On days 14–35, the MYCO treatment was lower than CONT, demonstrating the effect of mycotoxins, which was also observed in the HP+MYCO treatment when compared to HP. Furthermore, on days 14–35, when comparing the treatments that consumed SDPP and HP in the first two weeks, the animals that consumed SDPP had higher ADG (*p* < 0.05).

The results of daily feed intake (DFI) are presented in Table 2. On days 1–7 and 1–14, a higher DFI was observed for HP compared to CONT (*p* < 0.05). On days 1–14 and 8–14, the FI was higher in SDPP compared to CONT (*p* < 0.05). In the other treatments, there was no difference in FI, even when comparing HP and SDPP (*p* > 0.05). Regarding the feed conversion (FCR) presented in Table 2, it was found that on days 8–14, SDPP+MYCO had a higher FCR when contrasted with SDPP (*p* < 0.05).

There was an effect on the leukocyte count (*p* < 0.05; Table 3). When comparing CONT versus MYCO, the MYCO group had a lower leukocyte count (*p* < 0.05). HP group had a higher lymphocyte count compared to SDPP. When contrasted MYCO versus CONT, SDPP versus SDPP+MYCO, and MYCO versus HP+MYCO, the MYCO SDPP+MYCO treatments showed lower neutrophil counts. Animals that ingested mycotoxins (SDPP+MYCO) had higher erythrocyte, hemoglobin, and hematocrit levels compared to SDPP (*p* < 0.05). There was no effect on the contrasts for monocyte, eosinophil, and platelet counts (Table 3).

**Table 3:** Effect of treatment on hematological variables of nursery piglets receiving diets contaminated by mycotoxins and dehydrated swine plasma (SDPP) and hydrolysate of this plasma (HP)

Variables	CONT	MYCO	HP	HP+MYCO	SDPP	SDPP+MYCO	SEM	C1	C2	C3	C4	C5	C6	C7	C8
Leukocyte (×10^3^ µL)	14.8	12.5	16.6	14.7	15.5	13.2	0.83	0.05	0.09	0.18	0.08	0.87	0.07	0.82	0.76
Lymphocytes (×10^3^ µL)	7.43	7.13	9.24	7.96	6.94	7.45	0.42	0.93	0.1	0.83	0.08	0.79	0.96	0.91	0.03
Neutrophils (×10^3^ µL)	6.8	4.82	6.79	6.15	8.13	5.29	0.54	0.01	0.88	0.01	0.95	0.15	0.05	0.59	0.29
Monocytes (×10^3^ µL)	0.41	0.4	0.42	0.39	0.21	0.22	0.07	0.97	0.96	0.98	0.96	0.14	0.97	0.17	0.09
Eosinophils (×10^3^ µL)	0.23	0.21	0.2	0.32	0.23	0.27	0.04	0.92	0.35	0.91	0.93	0.95	0.71	0.89	0.94
Erythrocytes (×10^6^ µL)	6.55	6.93	6.11	6.25	5.93	7.24	0.18	0.91	0.93	0.05	0.83	0.77	0.88	0.67	0.92
Hemoglobin (g/dl)	10.9	11.5	10.9	11.4	10.5	13.1	0.29	0.85	0.81	0.05	0.89	0.94	0.98	0.22	0.93
Hematocrit (%)	40.3	41.3	39	40	37.6	44.9	0.89	0.92	0.96	0.04	0.97	0.36	0.94	0.55	0.46
Platelets (×10^3^ µL)	509	486	521	535	466	426	30.2	0.51	0.86	0.82	0.79	0.24	0.12	0.32	0.06

Data were analyzed by orthogonal contrasts, with the probability (*p*-value) described within the table: C1 (Cont ×Myco), C2 (HP x HP+Myco), C3 (SDPP x SDPP+Myco), C4 (Cont ×HP), C5 (Cont ×SDPP), C6 (Myco ×HP+Myco), C7 (Myco ×SDPP+Myco), C8 (HP x SDPP). Note: Each group had 6 repetitions (*n* = 6), where the bay average was used. SEM = standard error

**Fig. 1:**
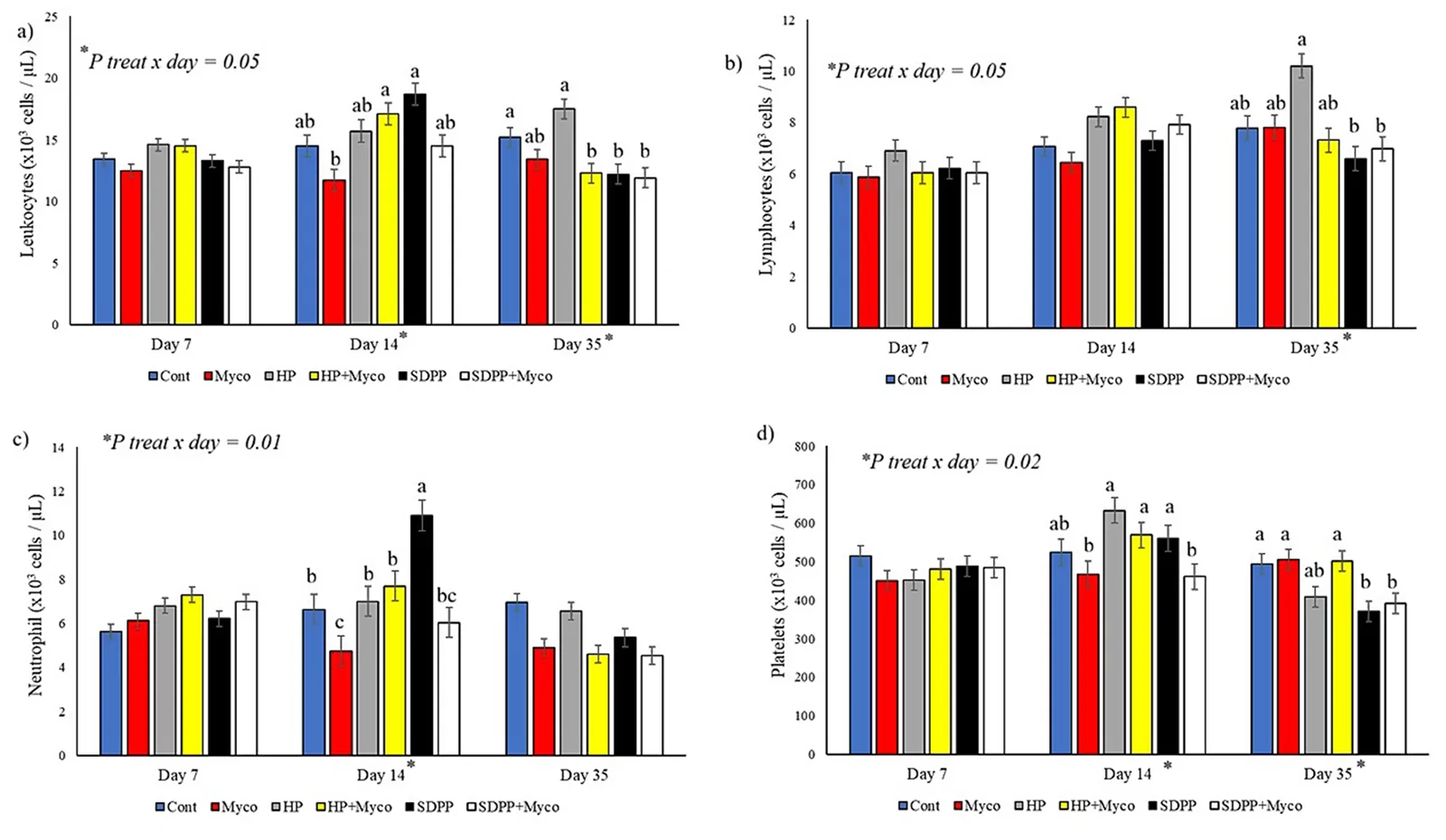
Treatment × day interaction for hematological variables of piglets exposed to a diet contaminated by mycotoxins and dehydrated swine plasma (SDPP) and plasma hydrolysate (HP). Note: y) the day the interaction occurred was marked with an asterisk (*), with the difference between the groups illustrated by different letters (a, b, c) above the bar. z) each group had 6 repetitions (*n* = 6), where the bay average was used

On day 14, MYCO group differed from HP+MYCO and SDPP, and had the lowest leukocyte count, but did not differ from CONT, HP, and SDPP+MYCO (Fig. 1a). On day 35, the HP+MYCO, SDPP, and SDPP+MYCO treatments had higher leukocyte counts compared to CONT, but did not differ from each other (Fig. 1a). On day 35, HP had higher lymphocyte counts than SDPP and SDPP+MYCO. However, HP, SDPP, and SDPP+MYCO did not differ from CONT, MYCO, and HP+MYCO (Fig. 1b). Compared to the control (CONT), on day 14 a lower neutrophil count was observed in the piglets of the MYCO group; as well as a higher count of this cell in the animals of the SDPP group (Fig. 1c).

In the contrast analysis, there was no treatment effect (Table 3); however, there was a treatment × day interaction (Fig. 1d). On day 14, the lowest platelet count was observed in animals from the MYCO and SDPP+MYCO groups when compared to three other groups (HP, HP+MYCO, and SDPP). On day 35, the lowest platelet count occurred in animals from the SDPP and SDPP+MYCO groups compared to the control group (Fig. 1d).

**Fig. 2:**
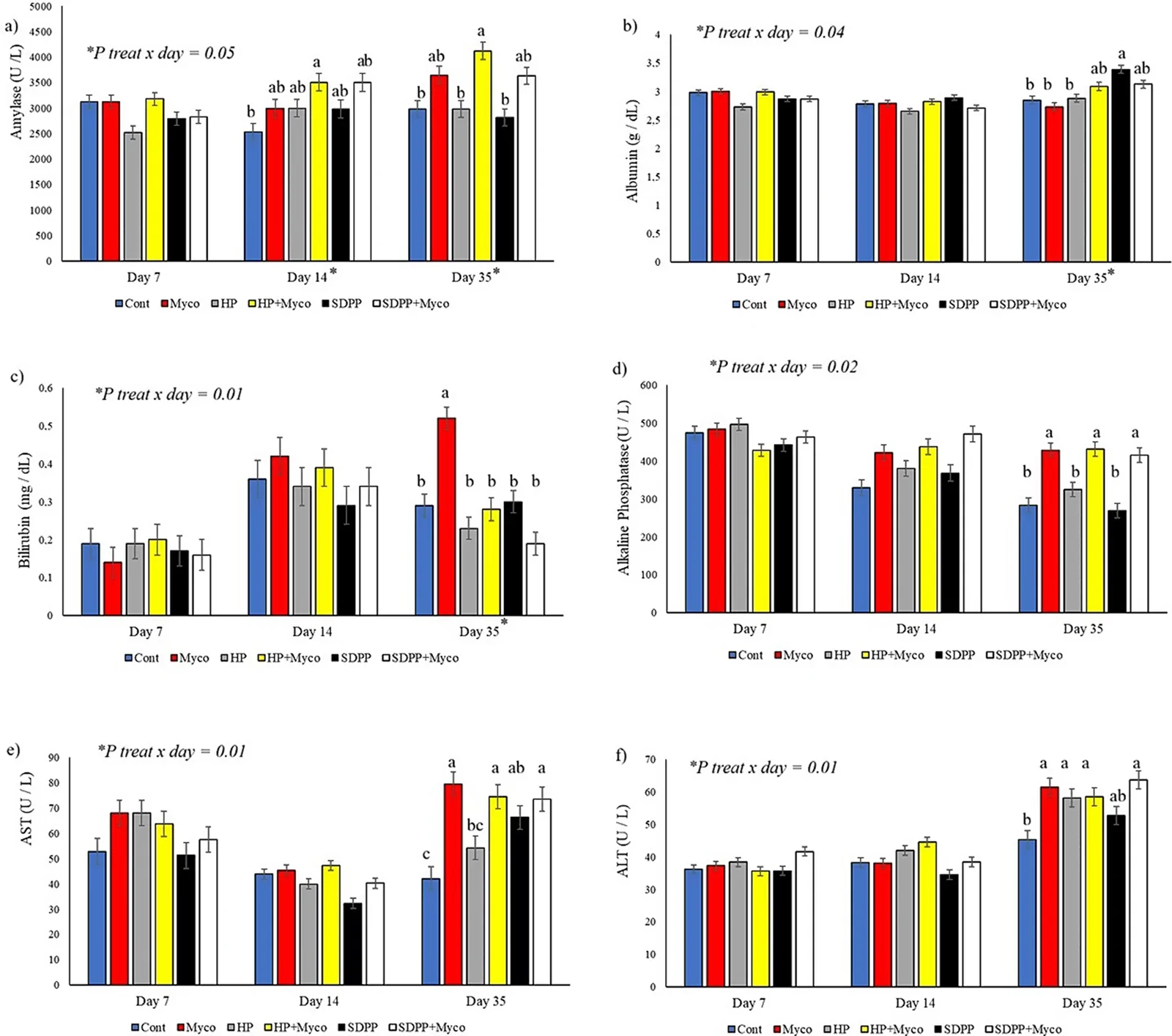
Treatment × day interaction on biochemical variables related pancreatic and hepatic health of piglets exposed to a diet contaminated by mycotoxins and dehydrated swine plasma (SDPP) and plasma hydrolysate (HP). Note: y) the day the interaction occurred was marked with an asterisk (*), with the difference between the groups illustrated by different letters (**a, b, c**) above the bar. z) each group had 6 repetitions (*n* = 6), where the bay average was used

Serum amylase activity was higher in the mycotoxin treatments when comparing CONT versus MYCO, HP versus HP+MYCO, and SDPP versus SDPP+MYCO (Table 4), while the results of the treatment × day interaction were presented in Fig. 2a. When comparing the SDPP and HP treatments, albumin levels were higher in the SDPP treatment (Table 4), and the results of the treatment × day interaction were presented in Fig. 2b. Serum bilirubin levels were higher in the MYCO treatment when compared to CONT, and in the SDPP+MYCO treatment compared to SDPP; as well as date of the treatment × day interaction were presented in Fig. 2c. Alkaline phosphatase enzyme activity was higher in the MYCO, HP+MYCO, and SDPP+MYCO treatments when comparing CONT versus MYCO, HP versus HP+MYCO, and SDPP versus SDPP+MYCO (Table 4), while the results of the treatment × day interaction were presented in Fig. 2d. AST and ALT enzyme activities were higher in the MYCO treatment when comparing CONT versus MYCO (Table 4), and the results of the treatment × day interaction were showed in Fig. 2e and f.

**Table 4:** Effect of treatment on biochemical, immunological and oxidative variables of nursery piglets receiving diets contaminated by mycotoxins (myco), dehydrated porcine plasma (SDPP) and hydrolysate of this plasma (HP)

Variable	CONT	MYCO	HP	HP+MYCO	SDPP	SDPP+MYCO	SEM	C1	C2	C3	C4	C5	C6	C7	C8
*Biochemistry*															
Albumin, g/dL	2.81	2.76	2.77	2.95	3.14	2.92	0.06	0.88	0.82	0.71	0.91	0.45	0.69	0.72	0.02
Amylase, U/L	2753	3321	2989	3813	2898	3568	141	0.05	0.02	0.04	0.86	0.89	0.77	0.85	0.94
Bilirubin, mg/dL	0.32	0.47	0.28	0.33	0.25	0.26	0.03	0.02	0.86	0.97	0.92	0.95	0.09	0.01	0.9
Alkaline phosphatase, U/L	307	425	353	435	318	444	19.9	0.01	0.02	0.01	0.32	0.93	0.94	0.88	0.64
AST, U/L	43	62.5	47.1	60.9	46.3	57.1	3.35	0.01	0.25	0.21	0.95	0.96	0.96	0.88	0.98
ALT, U/L	41.9	54.1	50.1	51.6	43.6	51.1	2.17	0.05	0.96	0.11	0.1	0.94	0.93	0.94	0.96
Glucose, mg/dL	111	116	116	115	121	114	2.39	0.95	0.96	0.81	0.89	0.87	0.96	0.95	0.79
Total Protein, g/dL	4.33	4.53	4.35	4.66	4.45	4.46	0.07	0.65	0.74	0.96	0.95	0.84	0.92	0.91	0.89
*Immunology*															
Creatine kinase, U/L	2285	1784	1913	1770	2765	2301	267	0.45	0.79	0.87	0.52	0.78	0.94	0.21	0.05
Globulin, g/dL	1.51	1.77	1.58	1.71	1.3	1.54	0.06	0.78	0.85	0.88	0.91	0.76	0.94	0.42	0.72
IgG, g/dL	97.7	107	99.1	122	93	123	4.15	0.35	0.05	0.03	0.97	0.91	0.36	0.33	0.92
IgA, g/dL	2.98	5.97	2.52	6.08	3.36	5.22	0.68	0.05	0.04	0.34	0.95	0.93	0.94	0.85	0.25
Transferrin, mg/dL	1.9	1.85	1.43	2.38	1.8	2.59	0.17	0.95	0.03	0.12	0.65	0.97	0.24	0.28	0.91
C-reactive Protein, mg/dL	0.53	0.58	0.52	0.64	0.69	0.51	0.04	0.75	0.26	0.03	0.98	0.04	0.82	0.79	0.04
Cholinesterase, U/L	673	809	744	810	692	823	29.1	0.02	0.21	0.01	0.42	0.89	0.94	0.92	0.55
*Oxidative status*															
ROS, U/fluorescence	13.4	18.9	18.8	19.1	13.4	17.4	1.24	0.05	0.96	0.07	0.05	0.97	0.89	0.85	0.05
TBARS, nmol/mL	28.8	43.6	46.6	37.1	37.8	47.2	4.89	0.05	0.52	0.63	0.05	0.19	0.87	0.89	0.62
MPO, U/L	3.22	6.77	3.45	5.85	4.94	6.00	0.87	0.01	0.05	0.54	0.76	0.26	0.84	0.86	0.33
PSH, µmol/L	2.51	4.24	2.31	3.57	3.27	3.93	0.41	0.02	0.09	0.12	0.91	0.49	0.66	0.84	0.39

Data were analyzed by orthogonal contrasts, with the probability (*p*-value) described within the table: C1 (Cont ×Myco), C2 (HP x HP+Myco), C3 (SDPP x SDPP+Myco), C4 (Cont ×HP), C5 (Cont ×SDPP), C6 (Myco ×HP+Myco), C7 (Myco ×SDPP+Myco), C8 (HP x SDPP). Note: Each group had 6 repetitions (*n* = 6), where the bay average was used. SEM = standard error

Serum amylase was higher in the HP+MYCO treatment compared to CONT; but did not differ from the other treatments. On day 35, HP+MYCO presented higher serum amylase levels than CONT, HP, and SDPP, but did not differ from MYCO and SDPP+MYCO (Fig. 2a). On day 35, SDPP showed higher serum albumin levels than CONT, MYCO, and HP, and did not differ from SDPP+MYCO and HP+MYCO (Fig. 2b). On day 35, bilirubin levels were higher in MYCO compared to the other treatments (Fig. 2c). On day 35, higher AP activity was observed in the serum of piglets that consumed mycotoxin (MYCO, HP+MYCO, and SDPP+MYCO) compared to the other groups (Fig. 2d). On day 14, SDPP and SDPP+MYCO presented lower AST enzyme activity than CONT, MYCO, and HP+MYCO (*p* < 0.05), but did not differ from HP group. On day 35, CONT animals differed from the other treatments, with the exception of HP, which presented the lowest AST enzyme activity (Fig. 2e). Animals in the MYCO, HP, HP+MYCO, and SDPP+MYCO groups showed higher ALT activity on day 35 compared to the CONT group (Fig. 2f).

**Fig. 3:**
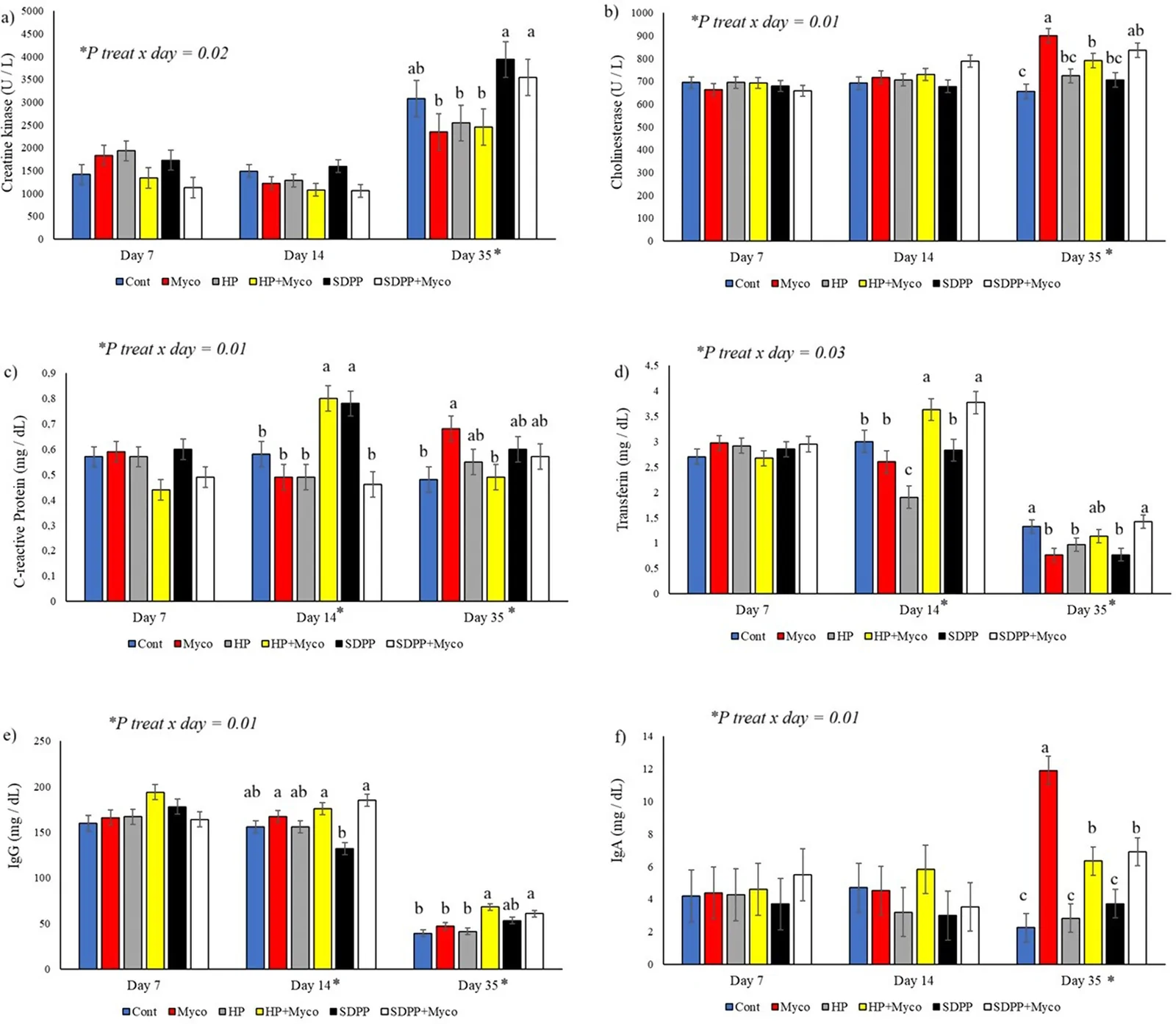
Treatment × day interaction on immunological variables of piglets exposed to a diet contaminated by mycotoxins and dehydrated swine plasma (SDPP) and plasma hydrolysate (HP). Note: y) the day the interaction occurred was marked with an asterisk (*), with the difference between the groups illustrated by different letters (**a, b, c**) above the bar. z) each group had 6 repetitions (*n* = 6), where the bay average was used

Creatine kinase was higher in the SDPP treatment compared to HP (Table 4), while the results of the treatment × day interaction were showed in Fig. 3a. When SDPP and SDPP+MYCO treatments were contrasted, SDPP+MYCO had the highest levels of total cholinesterase (Table 4), as well as the results of the treatment × day interaction were presented in Fig. 3b. For C-reactive protein (CRP), there was a difference between the SDPP and SDPP+MYCO, CONT and SDPP; and SDPP and HP contrasts, with the SDPP treatment having the highest CRP levels (Table 4), while the results of the treatment × day interaction were presented in Fig. 3c. For transferrin levels, there was a difference only in the contrast between HP and HP+MYCO, with the HP+MYCO treatment having the highest levels of this acute phase protein (Table 4), and the results of the treatment × day interaction were presented in Fig. 3d. When comparing the SDPP and SDPP+MYCO, HP and HP+MYCO treatments, the SDPP+MYCO and HP+MYCO mycotoxin treatments showed higher IgG levels; as well as the results of the treatment × day interaction were presented in Fig. 3e. For IgA, there were differences in the contrasts between CONT and MYCO, HP, and HP+MYCO, with the treatments containing mycotoxins MYCO and HP+MYCO presenting the highest IgA levels (Table 4); and the results of the treatment × day interaction were presented in Fig. 3f.

When animals consumed SDPP at day 35, in the presence or absence of mycotoxin, CK activity was higher in the serum of these animals (Fig. 3a). On day 35, higher cholinesterase activity was observed in the serum of animals that did not consume mycotoxin compared to the group that consumed alfatoxin and fumonisin (Fig. 3b). On day 14, C-reactive protein levels were higher in piglets from the HP+MYCO and SDPP groups; on day 35, they were higher in the MYCO group compared to the CONT and HP+MYCO groups (Fig. 3c). Transferrin levels were higher in the serum of piglets from the HP+MYCO and SDPP+MYCO groups compared to the other groups on day 14; while on day 35, lower levels of this acute-phase protein were observed in animals from the MYCO, HP, and SDPP groups compared to the CONT group (Fig. 3d). Lower IgG levels were seen in the serum of the SDPP group compared to the others on day 14; unlike what was observed on day 35, when higher levels were observed in the HP+MYCO and SDPP+MYCO groups compared to the other groups (Fig. 3e). Also on day 35, higher IgA levels were observed in the serum of the three groups of piglets that consumed mycotoxin compared to the three unchallenged groups (Fig. 3f); However, it is worth highlighting that IgA levels were higher in the MYCO group compared to the HP+MYCO and SDPP+MYCO groups.

**Fig. 4:**
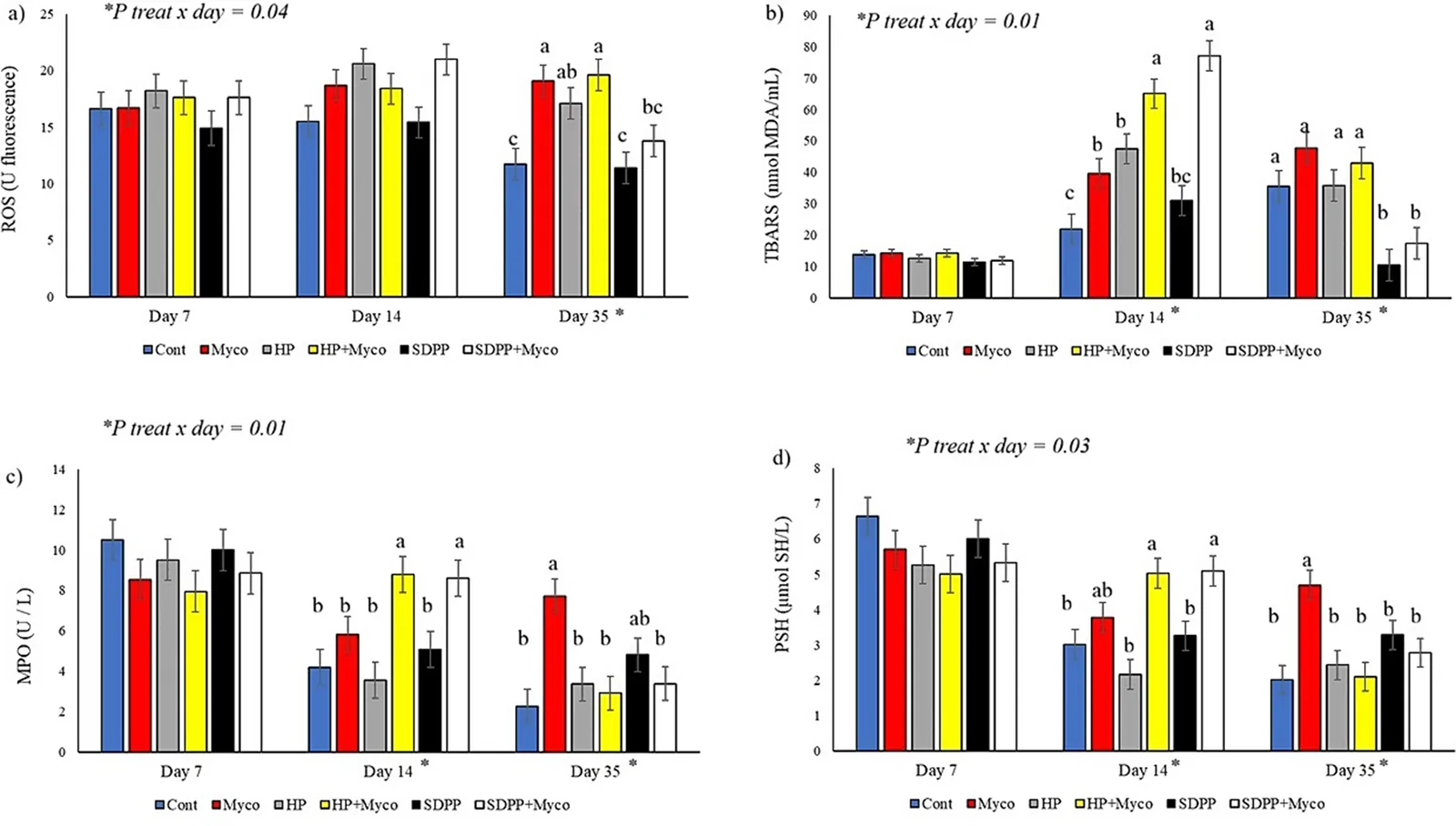
Treatment × day interaction on oxidative variables of piglets exposed to a diet contaminated by mycotoxins and dehydrated swine plasma (SDPP) and plasma hydrolysate (HP). Note: y) the day the interaction occurred was marked with an asterisk (*), with the difference between the groups illustrated by different letters (**a, b, c**) above the bar. z) each group had 6 repetitions (*n* = 6), where the bay average was used

When compared to CONT, the MYCO and HP treatments had higher levels of ROS and TBARS (Table 4); while the results of the treatment × day interaction were showed in Fig. 4 (a,b). When contrasted between CONT and MYCO, HP, and HP+MYCO, MPO (myeloperoxidase) levels were higher in the MYCO and HP+MYCO treatments (Table 4); and the results of the treatment × day interaction were presented in Fig. 4c. When contrasted between CONT and MYCO, the MYCO treatment had higher levels of PSH (protein thiols) (Table 4), similar results observed at treatment × day interaction (Fig. 4d). In all treatments, with the exception of HP+MYCO, SDPP was lowest on day 14, and there was no difference on day 35. However, for the HP+MYCO treatment, it was lowest only on day 35 (*p* < 0.05). An interaction between day and treatment was also observed on PSH levels on days 14 and 35 (Fig. 4). The MYCO, HP+MYCO, SDPP, and SDPP+MYCO treatments had higher PSH levels than CONT and HP, but there was no difference between them. On day 35, the MYCO and SDPP treatments had higher PSH levels than CONT, HP, HP+MYCO, and SDPP+MYCO (Fig. 4).

For the HP+MYCO and SDPP+MYCO treatments, levels were lower only on day 35. There was a day × treatment interaction on MPO levels (Fig. 4). On day 14, the HP+MYCO and SDPP+MYCO treatments presented the highest levels and did not differ from each other. HP, SDPP, and CONT did not differ from each other; and SDPP+MYCO did not differ from MYCO. On day 35, the MYCO and SDPP+MYCO treatments presented the highest levels and did not differ from each other. There was a treatment × day interaction (*p* < 0.05) for ROS and TBARS levels (Fig. 4). In the SDPP+MYCO treatment, there was a reduction in ROS levels on day 35 compared to day 14. The SDPP and SDPP+MYCO treatments did not differ from CONT, while the MYCO, HP, and HP+MYCO treatments showed higher ROS levels than CONT. On day 14, there was a difference between treatments (*p* < 0.05) for TBARS levels, in which the SDPP+MYCO and HP+MYCO treatments showed the highest levels, while the SDPP and HP treatments were higher than CONT, but did not differ from MYCO. On day 35, the SDPP and SDPP+MYCO treatments did not differ from each other; but differed from all the others and showed the lowest TBARS levels (Fig. 4).

## Discussion

Mycotoxins had a negative effect on piglet performance, compromising body weight, weight gain, feed intake, and feed conversion. Reduced performance under contamination conditions was expected and corroborates previous studies by our group (Müller et al. 2018a), and other published studies (Andretta et al. 2016; Yang et al. 2020; Wilson et al. 2022; Weaver et al. 2023; Tarasconi et al. 2024). Similar results were found by Holanda and Kim (2022), who tested diets containing 200 ppb of aflatoxins combined with 2 ppm of deoxynivalenol for piglets weaned at 28 days for 31 days. where the authors found that mycotoxins reduced the health and feed intake, daily weight gain, and body weight of piglets, in addition to presenting a tendency to elevate tumor necrosis factor in the intestine. It is already known that mycotoxins compromise the intestinal integrity of piglets (Alizadeh et al. 2015); reduce villus height, and consequently the absorptive capacity of the intestine (Kim et al. 2019); suppress the immune system, making them susceptible to infectious diseases (Pierron et al. 2016); contribute to the establishment of oxidative stress, endoplasmic reticulum stress, and cellular apoptosis (Yang et al. 2020); among other scientific findings. The sum of these harmful effects converges to reduce animal performance, since health status is directly related to productive capacity. Furthermore, Kipper et al. (2020) emphasizes that piglets are highly sensitive to the harmful effects of mycotoxins, especially when combined in the same diet, where their harmful effects are increased, either through synergism or additive effects. In this study, the negative effects of mycotoxins on weight gain were only observed during the period from day 15 to day 35 of the experiment, when feed consumption is higher per piglets; consequently, mycotoxin intake is also higher. These toxins have the characteristic of depositing in tissues and causing problems in animals due to the cumulative effect generated by daily consumption.

Adding SDPP to the diet increased weight gain and body weight compared to HP. Both SDPP and HP stimulated feed intake compared to the control in the first two weeks, which contained SDPP (6% in the first, and 4% in the second); however, there was no difference between them. The increase in feed intake and weight gain, resulting from the provision of SDPP, was also reported by Balan et al. (2021), through a meta-analysis, in which SDPP at weaning of piglets improved animal performance, especially in the first week.

Low intake in the first few days after weaning is one of the factors contributing to malnutrition, poor performance, and increased susceptibility to infections and diarrhea (Jayaraman and Nyachoti 2017). Therefore, when intake is stimulated, adequate nutrition consequently improves the health status and performance of animals. The improvement in intake from the provision of SDPP has already been observed by other researchers (Pérez-Bosque et al. 2016; Dos Santos Cerqueira et al. 2019; Castelo et al. 2023), including in situations of mycotoxin challenge (Müller et al. 2018a) and in situations of heat challenge (Dos Santos Cerqueira et al. 2019). The improvement in intake from SDPP and HP in the diet is probably due to the increased palatability of the feed, since increasing palatability is one of the strategies to improve piglet performance after weaning, especially in the first days of transition, when intake is normally significantly low (Wensley et al. 2021).

Mycotoxins increased serum amylase and bilirubin levels, alkaline phosphatase activity, when compared to other treatments, and AST and ALT activity, when compared to the control. AST and ALT activity are biomarkers used to indicate liver injury, as they are released when hepatocytes are ruptured or damaged (Gonzales and Silva, 2017). According to Dolenšek et al. (2021), mycotoxicosis can cause apoptosis and hepatocyte damage, as well as inflammatory infiltrates. Furthermore, scientific findings have found necrosis in cells of the mucosa of the stomach, duodenum, kidneys, and liver (Semenov et al. 2022). When liver cells are damaged or ruptured by the action of mycotoxins, they release the enzymes ALT and AST, which increases the activity of these enzymes and characterizes the toxicity of these molecules.

In our results, there was an increase in serum creatine kinase (CK) with SDPP. Baldissera et al. (2018) observed a correlation between serum creatine kinase levels and piglet body weight, in which higher CK activity were found in heavier animals. The findings of (He et al. 2018) corroborate this finding and the results of our study, because they observed that increasing CK leads to greater weight gain in pigs. The enzyme creatine kinase is a central regulator of cellular energy homeostasis, reversibly converting ADP to ATP (Aujla et al.). This could explain the high levels in the SDPP treatment, which also resulted in the highest body weights. Mycotoxins increased cholinesterase activity, an indicator of exposure to toxic compounds, as it is an enzyme that modulates the action of the neurotransmitter acetylcholine by deactivating it (Gonsalez and Silva, 2017). Mycotoxins also increased IgG and IgA levels; and this can be understood because mycotoxicosis modulate the immune system and increase the inflammation, because the increased production of some antibodies may also be a response to mycotoxin toxicity, as well as to opportunistic pathogens that can cause infections (Semenov et al. 2022).

The inclusion of HP in the diet increased neutrophil levels, even when challenged by mycotoxins, and presented higher lymphocyte levels compared to SDPP. These results they can suggest an immunomodulatory effect, with increased levels of defense cells. Treatments with SDPP showed reduced leukocyte levels. The same was true for lymphocyte levels, in which HP presented higher levels than SDPP. Furthermore, it provided a residual effect on lymphocyte levels, in which levels were higher on day 35 than on day 7. Regarding neutrophils, SDPP showed an increase, with the highest levels on day 14, with a significant reduction on day 35. Solà-Ginés et al. (2024) sought to characterize a SDPP hydrolysate and tested it in in vivo mouse models. They found that SDPP hydrolysate was capable of promoting antioxidant and immunomodulatory effects, with an increase in health status, based on its molecular properties. SDPP intake causes an increase of albumin levels compared to HP and prevented the increase in bilirubin under mycotoxin challenge conditions, demonstrating a hepatoprotective effect. Compared to HP and SDPP had higher CRP levels, and lower ROS levels. CRP is a highly sensitive inflammatory marker (Gonsalez and Silva, 2017), as well as the smaller amount of transferrin. Due to the larger and more complex plasma molecules compared to HP, this may have stimulated its production. The higher levels of ROS and TBARS in HP suggest that the hydrolysate somehow provides a pro-oxidant effect. This was unexpected, given that studies characterizing this same hydrolysate reported findings of peptides with antioxidant bioactivity in its composition (Solà-Ginés et al. 2024). SDPP presented a favorable antioxidant effect when contaminated with mycotoxins, and even with contamination, it had lower TBARS levels than the other treatments. Because when compared to the control, mycotoxins increased levels of ROS, TBARS, MPO, and PSH. The first three are indicators of oxidation, while PSH is an antioxidant protein thiol; i.e. all these parameters were elevated due to the effect of mycotoxins. Mycotoxicosis contributes to oxidative stress (Müller et al. 2018a). However, elevated antioxidant levels may be an adaptive response to enhance the body’s defense capacity (De Lourdes Pires Bianchi and Maria Greggi Antunes 1999). Protein thiols (PSH), which are active antioxidants (Gonsalez and Silva, 2017), were reduced on days 14 and 35 in all treatments. This can be explained by the stress caused by weaning, as weaning stress induces inflammatory processes and oxidative stress, dysfunctions in energy metabolism, and affects the immune system (Novais et al. 2021).

Mycotoxins increased MPO activity; however, when animals consumed SDPP and HP, this mycotoxin effect did not occur, and MPO activity did not differ from the control. HP increased the levels of immunological indicators. The effect of peptides on health was also reported by Shi et al. (2018), who also highlighted the antimicrobial bioactivities of some peptides. This corroborates the findings of Espinosa et al. (2022), who found that when both dehydrated bovine plasma and hydrolyzed bovine plasma were administered, there was a reduction in the incidence of diarrhea and an increase in piglet weight gain. González-Solé et al. (2020) also observed the effect of peptides on the expression of proteins involved in the immune response and suggested that both SDPP, and peptides would provide better immunological conditions in infectious processes and intestinal challenges. These findings corroborate our results, which suggest that HP intake has immunomodulatory capacity. However, the high levels of TBARS and ROS in HP compared to SDPP suggest a pro-oxidant action, but this effect needs to be investigated further in future studies. According to Soares et al. (2015), oxidative stress activates inflammatory processes, and vice versa. It may even have a feedback effect, in which one potentiates the other. Based on this principle, as HP increased oxidation parameters, but also had an effect on the immune system, it could be an associated effect.

## Conclusion

The addition of SDPP and HP to the mycotoxin-contaminated diet was unable to minimize or prevent the disorders caused by aflatoxin and fumonisin. The mycotoxins caused subclinical intoxication with alterations in sensitive biomarkers of cellular damage, and oxidative stress, in addition to compromising animal performance. SDPP group provided greater performance than HP group. The addition of HP stimulates feed consumption. SDPP and HP have different properties, which resulted in different results for various performance and health biomarkers in piglets under mycotoxin contamination. The plasma hydrolysis process modifies its properties.

## Electronic supplementary material

Below is the link to the electronic supplementary material.


Supplementary Material 1


## Data Availability

The data are with the authors and can be available upon request.

## References

[CR2] Ali SF, LeBel CP, Bondy SC (1992) Reactive oxygen species formation as a biomarker of methylmercury and trimethyltin neurotoxicity. Neurotoxicology 13:637–648 1475065

[CR3] Alizadeh A, Braber S, Akbari P, Garssen J, Fink-Gremmels J (2015) Deoxynivalenol impairs weight gain and affects markers of gut health after low-dose, short-term exposure of growing pigs. Toxins 7:2071–2095. 10.3390/toxins7062071 26067367 PMC4488690

[CR4] Andretta I, Kipper M, Hauschild L, Lehnen CR, Remus A, Melchior R (2016) Meta-analysis of individual and combined effects of mycotoxins on growing pigs. Scientia Agric 73:328–331. 10.1590/0103-9016-2015-0132

[CR7] Balan P, Staincliffe M, Moughan PJ (2021) Effects of spray-dried animal plasma on the growth performance of weaned piglets-A review. J Anim Physiol Anim Nutr (Berl) 105:699–714. 10.1111/jpn.13435 32860645

[CR8] Baldissera MD, Müller LKF, Souza CF, Santurio JM, Gloria EM, Machado G, Boiago MM, Paiano D, da Silva AS (2018) Creatine kinase and ATPase activities in piglets fed a fungal mycotoxin co-contaminated diet: consequences in the pathogenesis of subclinical intoxication. Microb Pathogen 122:13–18. 10.1016/j.micpath.2018.05.044 29852206

[CR13] Castelo PG, Rodrigues LA, de P GM, Guedes RMC, Moreno AM, Coura FM, Heinemann MB, Rosa BO, Brustolini APL, Araújo ICS, de O FD (2023) A dietary spray-dried plasma feeding programme improves growth performance and reduces faecal bacterial shedding of nursery pigs challenged with enterotoxigenic Escherichia coli K88. J Anim Physiol Anim Nutr 107:581–588. 10.1111/jpn.13761 35934921

[CR15] De Lourdes Pires Bianchi M, Maria Greggi Antunes L (1999) Free radicals and the main dietary antioxidants. Braz J Nutr 12:123–130

[CR16] Dolenšek T, Švara T, Knific T, Gombač M, Luzar B, Jakovac-Strajn B (2021) The influence of fusarium mycotoxins on the liver of gilts and their suckling piglets. Animals 11:2534. 10.3390/ani11092534 34573499 PMC8469335

[CR17] Dos Santos Cerqueira LG, Schinckel AP, Silveira H, Kuribayashi TH, Moreira RHR, de O LÍ, de S CV, Pospissil Garbossa CA (2019) Spray-dried porcine plasma improves feed intake of weaned piglets subjected to heat stress. J Anim Physiol Anim Nutr 103:836–845. 10.1111/jpn.13071 30775807

[CR18] Espinosa CD, Campbell JM, Stein HH (2022) Growth performance of weanling pigs fed diets containing spray-dried bovine plasma or hydrolyzed spray-dried bovine plasma. Anim Feed Sci Technol 294:115500. 10.1016/j.anifeedsci.2022.115500

[CR69] Feldman BF, Zinkl JG, Jain NC (2000) Schalm’s Veterinary Hematology. 5th ed. Philadelphia: Lippincott Williams & Wilkins

[CR72] Gonzales E, Silva SC (2017) Fisiologia das Aves Domésticas. 2. ed. Jaboticabal: FUNEP

[CR19] González-Solé F, Criado-Mesas L, Villodre C, García WC, Farré M, Borda E, Pérez-Cano FJ, Folch JM, Solà-Oriol D, Pérez JF (2020) Porcine digestible peptides (pdp) in weanling diets regulates the expression of genes involved in gut barrier function, immune response and nutrient transport in nursery pigs. Animals 10:1–21. 10.3390/ani10122368 PMC7763307 33321976

[CR20] He DT, Gai XR, Yang LB, Li JT, Lai WQ, Sun XL, Zhang LY (2018) Effects of guanidinoacetic acid on growth performance, creatine and energy metabolism, and carcass characteristics in growing-finishing pigs. J Anim Sci 96:3264–3273. 10.1093/jas/sky186 29741632 PMC6095271

[CR21] Heim G, Sweeney T, O’Shea CJ, Doyle DN, O’Doherty J (2015) Effect of maternal dietary supplementation of laminarin and fucoidan, independently or in combination, on pig growth performance and aspects of intestinal health. Anim Feed Sci Technol 204:28–41. 10.1016/j.anifeedsci.2015.02.007

[CR22] Holanda DM, Kim SW (2022) Impacts of weaning weights and mycotoxin challenges on jejunal mucosa-associated microbiota, intestinal and systemic health, and growth performance of nursery pigs. J Anim Sci Biotechnol 13:43. 10.1186/s40104-022-00691-6 35413935 PMC9006406

[CR25] Huting AMS, Middelkoop A, Guan X, Molist F (2021) Using nutritional strategies to shape the gastro-intestinal tracts of suckling and weaned piglets. Animals 11:1–37. 10.3390/ani11020402 PMC7914898 33562533

[CR26] Jayaraman B, Nyachoti CM (2017) Husbandry practices and gut health outcomes in weaned piglets: a review. Anim nutr 3:205–211. 10.1016/j.aninu.2017.06.002 29767154 PMC5941228

[CR27] Jentzsch AM, Bachmann HP, fürst HK (1996) Biesalski, improved analisys of malondialdehyde in human body fluids. Free Radic Biol Med 20:251–256 8746446 10.1016/0891-5849(95)02043-8

[CR31] Kim SW, Holanda DM, Gao X, Park I, Yiannikouris A (2019) Effcacy of a yeast cellwall extract to mitigate the effect of naturally co-occurring mycotoxins contaminating feed ingredients fed to young pigs: impact on gut health, microbiome, and growth. Toxins 11:633. 10.3390/toxins11110633 31683617 PMC6891535

[CR32] Kipper M, Andretta I, Ribeiro AML, da S PPG, Franceschina CS, Cardinal KM, de O MP, Schroeder B (2020) Assessing the implications of mycotoxins on productive efficiency of broilers and growing pigs. Scientia Agric 77. 10.1590/1678-992x-2018-0236

[CR33] Kolawole O, Siri-Anusornsak W, Petchkongkaew A, Elliott C (2024) A systematic review of global occurrence of emerging mycotoxins in crops and animal feeds, and their toxicity in livestock. Emerg Contam 10:100305. 10.1016/j.emcon.2024.100305

[CR71] Liew W-P-P, Mohd-Redzwan S (2018) Mycotoxin: its impact on gut health and microbiota. Front Cell Infect Microbiol, 8:60. 10.3389/fcimb.2018.00060 PMC5834427 29535978

[CR37] Maciag SS, Bellaver FV, Bombassaro G, Haach V, Morés MAZ, Baron LF, Coldebella A, Bastos AP (2022) On the influence of the source of porcine colostrum in the development of early immune ontogeny in piglets. Sci Rep 12:15630. 10.1038/s41598-022-20082-1 36115917 PMC9482628

[CR38] Mannervik B, Guthenberg C (1981) Glutathione transferase (human placenta). Methods Enzymol 77:231–235 7329301 10.1016/s0076-6879(81)77030-7

[CR41] Müller LKF, da Silva AS, Baldissera MD, Santurio JM, Glombowsky P, Gugel J, Campigotto G, Gloria EM, Paiano D, Machado G (2017) Effects of supplementation with spray-dried porcine plasma on blood variables on piglets feed with diet contaminated by mycotoxins. Microb Pathogen 110:464–470. 10.1016/j.micpath.2017.07.028 28733026

[CR43] Müller LKF, Paiano D, Bottari NB, Santurio JM, Zampar A, Schetinger MRC, Zanette RA, Mendes RE, Gloria EM, Baldissera MD, Silva DA, AS (2018a) Spray-dried porcine plasma added to diets contaminated with aflatoxin and fumonisins shows beneficial effects to piglet health. Anais Da Academia Brasileira de Cienc 90:3115–3128. 10.1590/0001-3765201820170794 30304239

[CR44] Müller LKF, Paiano D, Gugel J, Lorenzetti WR, Santurio JM, de Castro Tavernari F, da Gloria EM, Baldissera MD, Da Silva AS (2018b) Post-weaning piglets fed with different levels of fungal mycotoxins and spray-dried porcine plasma have improved weight gain, feed intake and reduced diarrhea incidence. Microb Pathogen 117:259–264. 10.1016/j.micpath.2018.02.035 29471136

[CR73] Moreno AM, Sobestiansky J, Lopez AC, Sobestiansky AAB (1997) Colheita e processamento de amostras de sangue em suínos para fins de diagnóstico. Embrapa Suínos e Aves 41:1–31

[CR45] Novais AK, Deschêne K, Martel-Kennes Y, Roy C, Laforest JP, Lessard M, Matte JJ, Lapointe J (2021) Weaning differentially affects mitochondrial function, oxidative stress, inflammation and apoptosis in normal and low birth weight piglets. PLoS One 16:e0247188. 10.1371/journal.pone.0247188 PMC7894895 33606751

[CR47] Pierron A, Alassane-Kpembi I, Oswald IP (2016) Impact of mycotoxin on immune response and consequences for pig health. Anim nutr 2:63–68. 10.1016/j.aninu.2016.03.001 29767037 PMC5941016

[CR46] Pérez-Bosque A, Polo J, Torrallardona D (2016) Spray dried plasma as an alternative to antibiotics in piglet feeds, mode of action and biosafety. Porcine Health Manag 2:16. 10.1186/s40813-016-0034-1 28405442 PMC5382520

[CR49] Rostagno HS, Albino LFT, Calderano AA, Hannas MI, Sakomura NK, Perazzo FG, Rocha GC, Saraiva A, Abreu MLT, Genova JL, Tavernari FC (2024) Tabelas Brasileiras para Aves e Suínos: composição de alimentos e exigências nutricionais. 5. ed. Visconde do Rio Branco: Suprema/Scienza, 531

[CR50] Sakomura NK (2014) Nutrição de não ruminantes. Funep, Jaboticabal, SP

[CR52] Sedlak R, Lindsay H (1968) Estimation of total, protein-bound, and nonprotein sulfhydryl groups in tissue with Ellman’s reagent. Analytical Biochemv 25:192–205 10.1016/0003-2697(68)90092-4 4973948

[CR53] Semenov EI, Matrosova LE, Tanaseva SA, Valiev AR, Potekhina RM, Tarasova EY, Spiridonov GN, Gubeeva EG, Mishina NN (2022) Experimental combined mycotoxicosis in pigs as affected by infection load. Sel’skokhozyaistvennaya Biologiya 57:371–383. 10.15389/agrobiology.2022.2.371eng

[CR54] Shi J, Zhang P, Meng XM, Fang Z, Lin Y, Che L, Feng B, Li J, Li G, Wu D, Xu S (2018) Effects of composite antimicrobial peptide on growth performance and health in weaned piglets. Anim Sci J 89:397–403. 10.1111/asj.12933 29082578

[CR55] Soares EDR, Monteiro EB, Da Silva RC, Batista A, Sobreira F, Mattos T, Da Costa CA, Daleprane JB (2015) Compostos bioativos em alimentos, estresse oxidativo e inflamação: uma visão molecular da nutrição. Revista Hosp Universitário Pedro Ernesto 14:64–72. 10.12957/rhupe.2015.19942

[CR56] Solà-Ginés M, Miró L, Bellver-Sanchis A, Griñán-Ferré C, Pallàs M, Pérez-Bosque A, Moretó M, Pont L, Benavente F, Barbosa J, Rodríguez C, Polo J (2024) Nutritional, molecular, and functional properties of a novel enzymatically hydrolyzed porcine plasma product. PLoS One 19:e0301504. 10.1371/journal.pone.0301504 PMC11086891 38728303

[CR70] Suzuki K, Ota H, Sasagawa S, Sakatani T, Fujikura T (1983) Assay method for myeloperoxidase in human polymorphonuclear leukocytes. Anal Biochem. 132(2):345–352. https://pubmed.ncbi.nlm.nih.gov/6312841/10.1016/0003-2697(83)90019-2 6312841

[CR57] Tang X, Xiong K, Fang R, Li M (2022) Weaning stress and intestinal health of piglets: a review. Front Immunol 13:1042778. 10.3389/fimmu.2022.1042778 36505434 PMC9730250

[CR58] Tarasconi L, Dazuk V, Molosse VL, Cécere BGO, Deolindo GL, Mendes RE, Gloria EM, Ternus EM, Galli GM, Paiano D, Da Silva AS (2024) Nursery pigs fed with feed contaminated by aflatoxin B1 (aspergillus flavus) and anti-mycotoxin blend: pathogenesis and negative impact on animal health and weight gain. Microb Pathogen 186:106474. 10.1016/j.micpath.2023.106474 38070627

[CR64] Weaver AC, Weaver DM, Adams N, Yiannikouris A (2023) Use of yeast cell wall extract for growing pigs consuming feed contaminated with mycotoxins below or above regulatory guidelines: a meta-analysis with meta-regression. Toxins 15:596. 10.3390/toxins15100596 37888627 PMC10611179

[CR62] Wensley MR, Tokach MD, Woodworth JC, Goodband RD, Gebhardt JT, Derouchey JM, McKilligan D (2021) Maintaining continuity of nutrient intake after weaning. II. Review of post-weaning strategies. Transl Anim Sci 5:1–16. 10.1093/tas/txab022 34841202 PMC8611789

[CR66] Wilson VC, Ramirez SM, Murugesan GR, Hofstetter U, Kerr BJ (2022) Effects of feeding variable levels of mycotoxins with or without a mitigation strategy on growth performance, gut permeability, and oxidative biomarkers in nursery pigs. Transl Anim Sci 6:1–7. 10.1093/tas/txac126 PMC9512096 36172461

[CR67] Yang C, Song G, Lim W (2020) Effects of mycotoxin-contaminated feed on farm animals. J Hazard Mater 389:122087. 10.1016/j.jhazmat.2020.122087 32004836

[CR68] Zhang S, Piao X, Ma X, Xu X, Zeng Z, Tian Q, Li Y (2015) Comparison of spray-dried egg and albumen powder with conventional animal protein sources as feed ingredients in diets fed to weaned pigs. Anim Sci J 86:772–781. 10.1111/asj.12359 25827306

