# Supplementary material for: Effect of spray-dried porcine plasma and plasma hydrolysate on the health and performance of mycotoxin-challenged piglets at weaning
Source: Trop Anim Health Prod. 2026 Feb 26;58(2):140. doi: 10.1007/s11250-026-04923-z (PMC12945970; doi:10.1007/s11250-026-04923-z)
Supplement: Supplementary file 1 — Supplementary Material 1 [file 11250_2026_4923_MOESM1_ESM.docx]

Table S1. Acquisition parameters data from mass spectrometer.

| **Analyte** | **MRM Transiction** | **Dwell Time (s)** | **Cone**  **Voltage (V)** | **Collision Energy (eV)** |
| --- | --- | --- | --- | --- |
| Aflatoxin B1 | 313.08>241.23 | 0.005 | 30 | 37 |
|  | 313.08>285.39 |  |  | 23 |
| IS Aflatoxin B1 | 330.00>300.90 | 0.005 | 30 | 23 |
| Aflatoxin B2 | 315.10>259.05 | 0.005 | 30 | 28 |
|  | 315.10>287.16 |  |  | 25 |
| IS Aflatoxin B2 | 332.00>303.10 | 0.005 | 30 | 25 |
| Aflatoxin G1 | 329.09>243.10 | 0.005 | 25 | 26 |
|  | 329.09>283.00 |  |  | 26 |
| IS Aflatoxin G1 | 346.00>257.00 | 0.005 | 25 | 26 |
| Aflatoxin G2 | 331.05>245.05 | 0.005 | 25 | 30 |
|  | 331.05>257.04 |  |  | 25 |
| IS Aflatoxin G2 | 348.00>330.00 | 0.005 | 25 | 25 |

| Fumonisin B1 | 722.78>334.29 | 0.005 | 30 | 40 |
| --- | --- | --- | --- | --- |
|  | 722.78>352.22 |  |  | 35 |
| IS Fumonisin B1 | 756.10>374.20 | 0.005 | 30 | 40 |
| Fumonisin B2 | 706.57>318.22 | 0.005 | 30 | 40 |
|  | 706.57>336.04 |  |  | 40 |
| IS Fumonisin B2 | 740.20>358.20 | 0.005 | 30 | 36 |

Table S2: Linearity of commercial kits used in the analyses, as well as reference range for piglets.

| Item | Linearity | Methods | Reference range^1^ |
| --- | --- | --- | --- |
| Albumin | < 6.0 g/dL | Colorimetric (Bromocresol Green), ~630 nm | 2.5 - 4.0 g/dL |
| Amylase | < 2.000 U/L | Colorimetric enzymatic, ~405 nm | - |
| Bilirubin | < 25 mg/dL | Colorimetric – Diazo (Jendrassik–Grof) | < 0.3 mg/dL |
| Creatine kinase | < 2000 U/L | UV Kinetic – IFCC (CK-NAC) | < 489 U/L |
| Alkaline phosphatase | < 500 U/L | Kinetic enzyme – IFCC (pNPP) | < 176 U/L |
| AST | < 400 U/L | UV Kinetics – IFCC | < 55 U/L |
| ALT | < 400 U/L | UV Kinetics – IFCC | < 46 U/L |
| Glucose | < 400 mg/dL | Enzymatic colorimetric | 66-116 mg/dL |
| Total Protein | < 14 g/dL | Colorimetric – Biuret | 5.0 – 7.0 g/dL |
| Globulin | - | Mathematically (total protein - albumin) | 5.3-6.4 g/dL |
| IgA | 5 – 800 mg/dL | Immunoturbidimetry | 2.0 – 12.0 mg/dL |
| C-reactive Protein | 2 – 160 mg/L | Immunoturbidimetry | < 1.0 mg/dL |
| Cholinesterase | < 20.000 U/L | Colorimetric kinetic enzyme | - |
| IgG | 34 – 3900 mg/dL | Immunoturbidimetry | 60.0 – 150.0 mg/dL |
| Transferrin | 6 – 650 mg/dL | Immunoturbidimetry | 2.0 – 4.0 mg/dL |
| Cholesterol | < 800 mg/dL | Enzymatic colorimetric | 60 - 120 mg/dL |

Note 1: Kaneko et al. (2008).
